# Plasma Orexin-A Levels Do Not Undergo Circadian Rhythm in Young Healthy Male Subjects

**DOI:** 10.3389/fendo.2018.00710

**Published:** 2018-12-05

**Authors:** Kari A. Mäkelä, Toni Karhu, Alicia Jurado Acosta, Olavi Vakkuri, Juhani Leppäluoto, Karl-Heinz Herzig

**Affiliations:** ^1^Research Unit of Biomedicine, Physiology, University of Oulu, Oulu, Finland; ^2^Biocenter Oulu, University of Oulu, Oulu, Finland; ^3^Oulu University Hospital and Medical Research Center Oulu, Oulu, Finland; ^4^Department of Gastroenterology and Metabolism, Poznan University of Medical Sciences, Poznan, Poland

**Keywords:** orexins, hypocretins, melatonin, cortisol, circadian rhythm

## Abstract

Orexin-A (OXA) has been originally isolated from a precursor peptide prepro-orexin from the lateral hypothalamus. The orexin system has been attributed to important functions in sleep, arousal and regulation of energy homeostasis. In addition to its high levels in cerebrospinal fluid, OXA is present in blood. However, reported peptide concentrations in plasma vary significantly depending on the method used. Therefore, a specific and sensitive OXA radioimmunoassay (RIA) with solid phase extraction method was developed to determine whether plasma OXA concentrations is affected by acute feeding and/or wake and sleep in young healthy males. Blood samples were collected for 24 h from nine healthy males (aged 20–24 years; BMI 20.7–26.5) every 2 h starting at 11 a.m. Food was served at 12 p.m, 5:30 p.m, 8 p.m and 8 a.m and the sleep time was between 10 p.m and 7 a.m. Plasma samples were analyzed in addition for cortisol and melatonin levels. Blood pressure was monitored through the experimental period. OXA antibody was raised in rabbits. OXA antiserum had only minor cross-reactivity with prepro-orexin precursor (<0.001%), amino-terminal peptide (<0.001%), carboxy-terminal peptide (0.001%), and orexin-B (0.3%) with high sensitivity (0.15 pg/tube). Plasma OXA levels varied between 0.5 and 16 pg/ml in seven subjects and were undetectable (below 0.5 pg/ml) in two subjects. The OXA concentrations did not correlate to feeding nor wake/sleep, whereas cortisol, melatonin and mean arterial blood pressure presented a clear circadian rhythm in each subject. In conclusion, OXA is present in blood in low amounts and its levels do not follow autonomic nor neuroendocrine circadian rhythms. Thereby, studies examining regulatory mechanisms and influences of OXA from blood samples should interpret results very cautiously.

## Introduction

Orexin-A and orexin-B, also called hypocretins, are peptides which were originally isolated from rat hypothalamus and are derived from the precursor peptide prepro-orexin (PPO) (1). Defects in orexin secretion result in narcolepsy in dogs, mice and humans, and orexins have been shown to have a role in stabilization of arousal (2–9). Currently, dual orexin receptor antagonists are commercially available or under investigation for treatment of insomnia (10). We have shown that transgenic mice overexpressing human PPO had reduced REM sleep compared with wild type mice indicating the importance of orexins in regulation of REM sleep and stabilization of arousal (11). In healthy humans, cerebrospinal fluid (CSF) OXA fluctuates depending on the time of the day with peak concentrations late in the night (3, 9).

Narcoleptic patients with cataplexy have low levels of OXA in their cerebrospinal fluid (6). They also have an altered secretion of melatonin with high daytime secretion, suggesting that orexin might have a role in the control of melatonin release (12). It has been shown that patients with insomnia have significantly elevated plasma OXA levels compared to healthy subjects (13). Furthermore, OXA is well-established for its actions on both short- and long-term energy homeostasis. In rodents, both acute and chronic central administration of OXA induces feeding (14–17). In humans, CSF OXA correlated negatively with body weight, but did not relate to body adiposity (18). Plasma OXA levels have been shown to correlate positively to BMI suggesting that orexins influence whole-body energy metabolism (1, 19). Finally, intermittent daily fasting increased plasma OXA levels during fasting hours in healthy male volunteers (20). However, it is unknown whether plasma OXA levels correlate to acute feeding and/or wakefulness and sleep in humans.

Immunoreactive OXA is present in human plasma, but its source for secretion is still unclear (21). In the brain, orexins are produced in the hypothalamus (14, 22, 23). Orexin-secreting neurons have dense projections throughout the central nervous system (CNS) (14, 22–27). Thus, the central secretion of orexins from the hypothalamus may influence nearby structures responsible in regulating food intake and sleep/wake, and possibly, others. In peripheral tissues, the presence of OXA and/or its receptors has been confirmed in the male reproductive organs, pancreas and adrenal gland (28–31). Previous studies reported significant variations in plasma immunoreactive OXA levels from lower than 1 pg/ml to around 2,500 pg/ml, and immunoreactivities in high performance liquid chromatography (HPLC) revealed two or more peaks (13, 19, 20, 25, 32–37). Therefore, assays with better specificity and sensitivity are required for improved reliability determination of circulating OXA.

CSF OXA shows a circadian rhythm with peak concentrations late in the night (3). Despite its potentially large clinical significance in humans, circadian rhythm of circulating OXA has not been studied in detail nor has it been examined adequately via blood plasma concentration. The purpose of the present study was to assess plasma OXA concentrations extracted by solid phase method in a healthy group over a complete circadian cycle via a novel specific and sensitive radioimmunoassay (RIA). The OXA levels were compared with timing of meals and sleep/wake rhythm and well-established circadian rhythms of melatonin and cortisol.

## Materials and Methods

### Subjects

Nine young healthy male volunteers with no anamnestic narcolepsy (aged 20–24 years; BMI 20.7–26.5) participated in the study. The experiment followed the standards set by the Declaration of Helsinki. The study protocol was approved by the institutional ethics committee (Northern Ostrobothnia Hospital District). All subjects gave their informed consent. The Municipal Board of ethics committee approved the trial under the registration number 14/2009.

### Experimental Protocol

No alcohol, smoking or heavy exercise was allowed 24 h before the study. Participants arrived at 9:00 a.m to the Institute. A catheter was inserted into the antecubital vein of the forearm. The testing facility offered computers, television and books/magazines for entertainment. Energy controlled food was available at 12 p.m (lunch: 4,426 kJ on average), 5:30 p.m (dinner: 3,206 kJ), 8 p.m (evening snack: 1,348 kJ), and 8 a.m (breakfast: 3,157 kJ). Lights were turned off at 10 p.m, and back on at 7 a.m. The volunteers were woken up for each blood sampling and continued sleeping thereafter. Blood samples were drawn and collected in 10 ml EDTA-coated tubes every 2 h starting at 11 a.m. Blood tubes were put immediately on ice, centrifuged at 4°C at 3,000 rpm for 10 min and the plasma was separated into tubes and stored in −70°C until analysis. Blood pressure was monitored throughout the study after each blood draw, except at 1 p.m, with an automatic blood pressure monitor (Omron M2, OMRON HEALTHCARE Co., Ltd., Kyoto, Japan).

### Preparation of Antisera and Tracer for OXA RIA

OXA antigen was prepared by coupling 2 mg of synthetic OXA (Orexin-A, Catalog# 003-30, Phoenix Pharmaceuticals, Burlingame, CA, USA) to 8 mg of bovine thyroglobulin (Cat. No. 89385, Sigma-Aldrich, St. Louis, MO, USA) by 100 mg carbodiimide (3-dimethyl-aminopropyl, Fluka, Switzerland) at (+4°C for 1 h). Two adult male rabbits weighing 4 kg were immunized with 0.5 mg of the conjugate in Freund's complete adjuvant (Difco Laboratories Inc, Detroit, MI, USA) and boosters of 0.3 mg were given at 6 and 10 weeks. At 12 weeks the rabbits were bled and the antisera collected. The rabbits had access to their regular rabbit diet and water *ad libitum*. The animals' pain and suffering was minimized during the experiment and euthanasia. The animal experiment was approved by the University of Oulu Ethical Committee for Animal Experiments and by the Regional Committee of the State Provincial Office. The animal experiment was performed according to the “legislation for the protection of animals used for scientific purposes” (the European Community Council Directive 86/609/EEC).

Antiserum from the rabbit 9/3 bound 40% of the tracer at the dilution of 1:500,000 and showed significant displacement of the tracer at the level of 0.015 pg OXA/tube. Cross-reactivity of OXB (Orexin B, Catalog# 003-31, Phoenix Pharmaceuticals) was 0.3% and that of PPO (Orexin, Prepro, Catalog# 003-45, Phoenix Pharmaceuticals), 1-15 OXA (Orexin A (1-15), Catalog# 003-49, Phoenix Pharmaceuticals) and 16-33 OXA amide (Orexin A (16-33) Amide, Catalog# 003-36, Phoenix Pharmaceuticals) <0.001%.

The tracer was prepared by radioiodination 0.5 m Ci Na^125^I to OXA (1 μg) by Chloramine T (10 μg) and purified in a 0.5 × 5 cm Sephadex G25 column eluted by 30% acetic acid followed by RP-HPLC on a Symmetry C_18_ column (4.6 × 150 mm, Waters, Milford, MA, USA). The column was eluted with a 30 min gradient of 20–50% acetonitrile in 0.1% trifluoroacetic acid.

### Radioimmunoassay of OXA

Calibrator solutions, samples and antisera were made up in RIA assay buffer (0.04 mol/l sodium hydrogen phosphate, 0.01 mol/l sodium dihydrogen phosphate, 0.1 mol/l NaCl, 1 g/l gelatin, 0.5 ml/l Triton X-100, pH 7.4). Duplicate calibrators (OXA 0.075–25 pg/tube), orexin related peptides (100–100,000 pg/tube) and dilutions of evaporated plasma samples were prepared. Tracer containing 5,000 counts per minute and antiserum at dilution 1:500,000 were incubated with calibrators or samples for 24–48 h at 4°C, each in volumes of 100 μl. The immunoglobulins were precipitated by centrifugation with donkey anti-rabbit IgG in 0.5 ml of 80 g/l polyethylene glycol 6,000 containing normal rabbit serum as a carrier.

### Solid Phase Extraction, gel Filtration, HPLC and RIA Validation of Plasma Samples

Human plasma samples (1 ml) were mixed with of 1 mol/l HCl (0.2 ml) containing 16 g/l glycine and submitted to solid phase extraction with Sep-Pak C_18_ cartridges in an automated Gilson Aspec system (Gilson, Middleton, WI, USA). After the loading the columns were washed with 1% acetonitrile and OXA was eluted by 2 ml of 80% acetonitrile in. The eluates were evaporated by SpeedVac (Savant Instruments Inc., Hicksville NY, USA) and the extracts were reconstituted with 250 μl of the RIA buffer.

Serial dilutions of extracted human plasma samples were parallel with OXA calibrators (data not shown). We determined recoveries by measuring plasma pools to which 25 or 50 pg synthetic OXA was added before solid phase extraction. In two separate experiments the recoveries for 25 pg of OXA were 57 and 62% (*n* = 8) and for 50 pg 60 and 65% (*n* = 8). The OXA levels in all the subjects were measured in duplicates in one assay with intra-assay variation of coefficient of 8.2%.

Evaporated solid phase extracts of human plasma (2–4 ml) were applied to Sephadex G50 fine column (0.9 × 50 cm) eluted with 30% acetic acid. Fractions of 1 ml were collected, evaporated and assayed for OXA and absorbance at 280 nm. OXA containing fractions were further submitted to RP-HPLC and assayed for OXA. Gel filtration and HPLC runs were also performed in samples in which 50 pg of synthetic OXA was added. Synthetic and plasma OXA eluted at 13–15 ml and was subjected to HPLC after lyophilization.

Four ml of human plasma was extracted with Sep-Pak C_18_ Cartridges and further subjected to HPLC. Elution profile was a linear gradient from 0 to 60% of acetonitrile in 0.1% trifluoroacetic acid. A flow-rate of 1 ml/run was used. Only one OXA immunoreactive peak was observed eluting as the synthetic OXA.

### Melatonin and Cortisol RIAs

Plasma melatonin levels were analyzed using an in-house RIA (38). Samples of 0.8–1 ml were extracted with 4 ml of chloroform. After the evaporation of the organic phase the samples were dissolved in 250 μl of PBS for RIA. From these samples, 100 μl fractions were assayed for melatonin RIA in duplicates. The intra-assay coefficient of variation was 7.4%.

Plasma cortisol was measured using a RIA assay according to manufacturer's instructions (Spectria, Orion Diagnostica Oy, Espoo, Finland). Plasma samples of 20 μl were used per well in duplicates. The intra-assay coefficient of variation was 5.7%.

### Statistical Analysis

Serial measurements of plasma OXA, cortisol and melatonin concentrations from each subject were analyzed using summary measures (39). In addition, we analyzed the data by repeated measures ANOVA. The data for cortisol and melatonin from every subject was analyzed using two different summarizing measures: height of peak and time to reach peak. Thus, the variations and locations in the shapes of the curves could be seen separately within each individual subject. The data for plasma OXA is presented with individual curves for each subject. The data for cortisol, melatonin and mean arterial pressure (MAP) is presented as mean ± standard error of mean (SEM). MAP was calculated using the following formula:

DBP + 1/3 (SBP–DBP), where DBP represents diastolic blood pressure and SBP represents systolic blood pressure.

## Results

**Figure 1:**
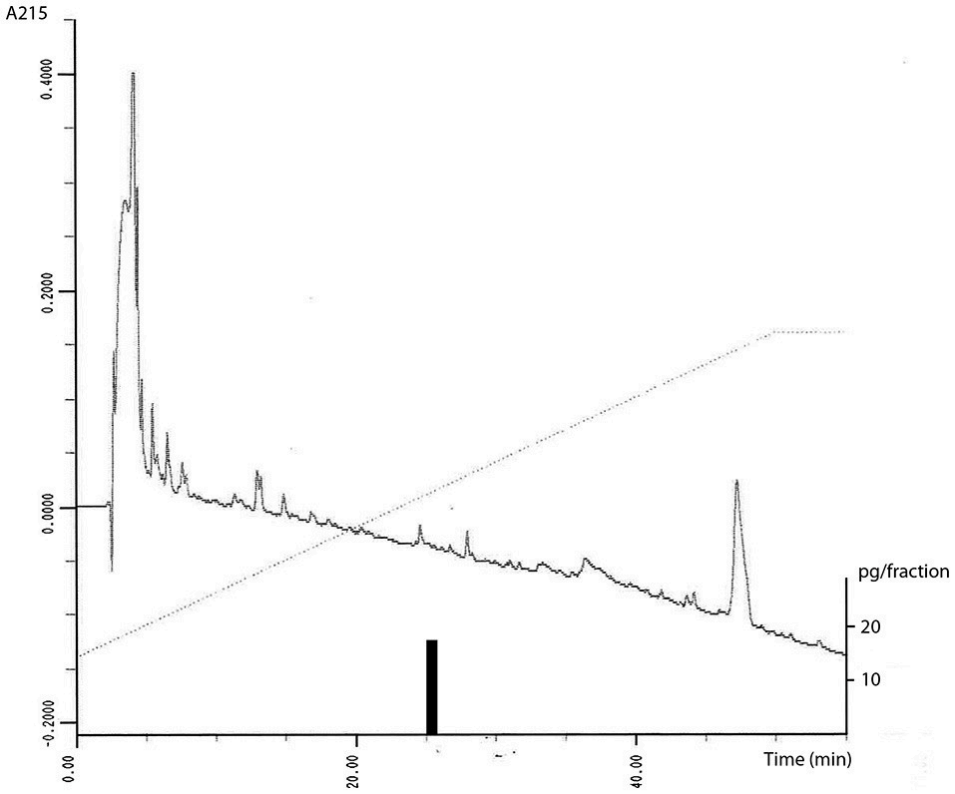
The figure depicts OXA concentrations in HPLC fractions of 4 ml human plasma extracted with Sep-Pak C_18_ Cartridges. Elution from 0 to 60% of acetonitrile in 0.1% trifluoroacetic acid is shown by a dotted line with flow-rate 1 ml/min. Only fraction number 26 contained (1 min fraction) 17 pg immunoreactive OXA (right vertical axis is added to show the pg/fraction and a black bar to indicate the amount of OXA).

The novel OXA assay used in this study had a high sensitivity of 0.15 pg of peptide/tube. OXA antiserum had minor cross-reactivity with PPO (<0.001%), aminoterminal peptide (<0.001%), and carboxyterminal peptide (0.001%). Antiserum showed 0.3% cross-reactivity with OXB. In RP-HPLC one immunoreactive peak from plasma extracts was observed eluting at 26 min. Synthetic OXA eluted at the same time (Figure 1).

**Figure 2:**
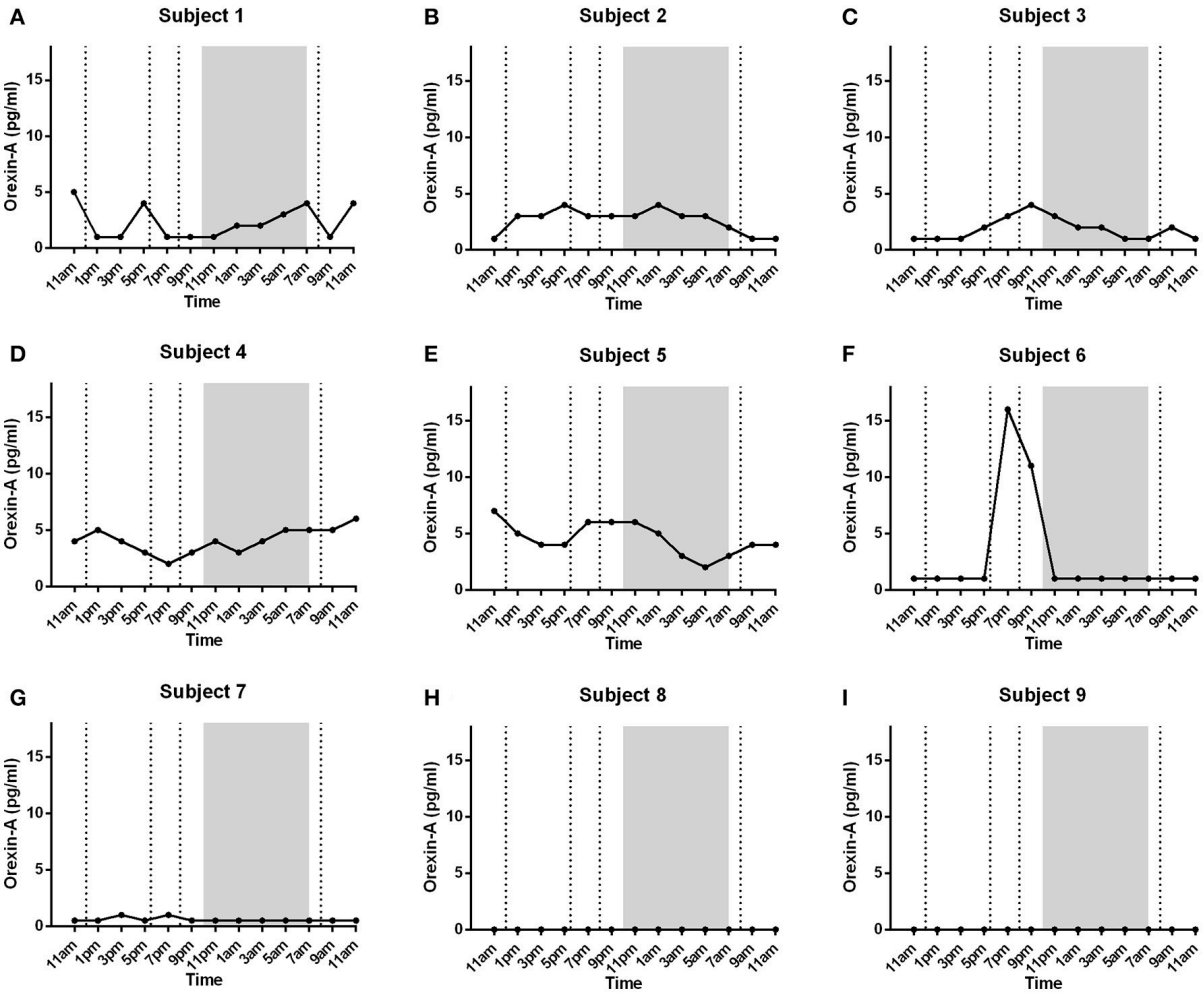
Individual data of plasma OXA levels in nine healthy male subjects (**(A-G)** and **(H,I)** were added to the text): Lunch, dinner, evening snack and breakfast are marked with dotted lines in respective order. Night-time (lights off) is marked with shaded region. Plasma concentrations varied from 0.5 – 16 pg/ml within 7 subjects **(A-G)** with no association to eating, wake or sleep between different subjects. In 2 subjects **(H,I)** plasma OXA levels were undetectable (below 0.5 pg/ml).

Diurnal plasma OXA concentrations varied greatly between subjects (Figures 2A–I). Five subjects had plasma OXA levels between 1 and 7 pg/ml (Figures 2A–E), while one subject showed high variations of OXA between the range of 1–16 pg/ml with a high peak between 7 and 9 p.m. (Figure 2F). One subject had low level of plasma OXA (0.5–1 pg/ml, Figure 2G), and the peptide was not detected (below 0.5 pg/ml) in two subjects (Figures 2H–I). OXA peaked once or more during the whole day within different subjects with no discernable pattern with eating, wake or sleep when compared between subjects. Repeated measures ANOVA did not show any significant changes (*p* = 0.4040) between the samples taken at different time points.

**Figure 3:**
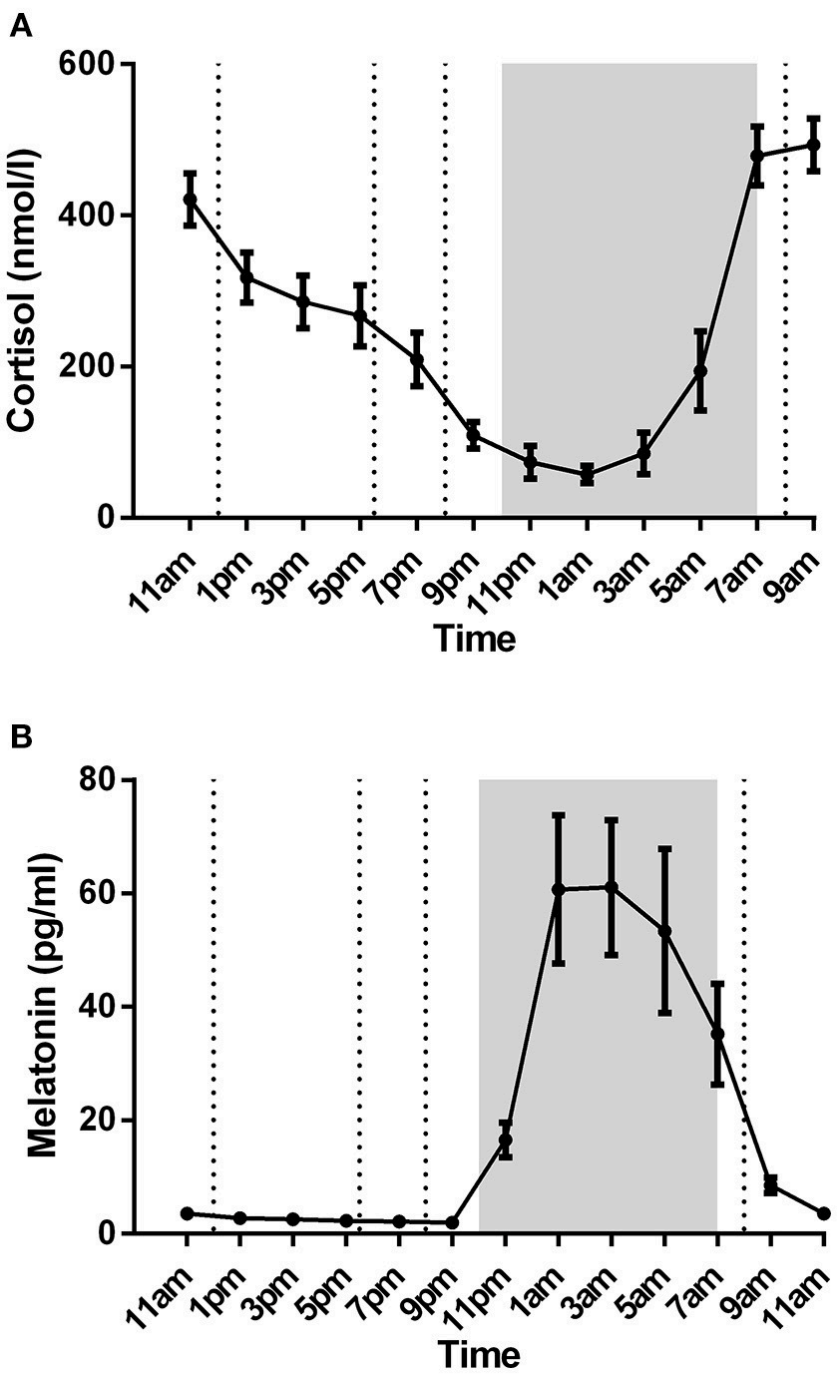
Plasma cortisol **(A)** and melatonin **(B)** levels of nine healthy male subjects (mean ± SEM). Lunch, dinner, evening snack, and breakfast are marked with dotted lines in respective order. Night-time (lights off at 10 p.m; lights on at 7 a.m) is marked with shaded region. Both cortisol and melatonin showed a normal circadian rhythm.

Plasma cortisol concentrations varied according to a normal daily cycle with decreasing levels toward the early morning and increasing levels toward the mid-morning (Figure 3A). Means from lowest/highest peaks as well as (clock) times to reach peaks were analyzed using individual summary data for each subject (data not shown). Cortisol levels were lowest (37.22 nmol/l) at 1.22 a.m (as calculated using summary measures; time to reach peak) whereas the highest peak (553mmol/l) was observed at 7.88 a.m. The cortisol concentrations of five subjects started to decrease after 7 a.m, while four subjects showed still high values at 9 a.m.

Plasma melatonin concentrations showed a clear circadian variation within all subjects with low concentrations during daytime and high concentrations during nighttime (Figure 3B). Daytime levels varied between 1.94 pg/ml (at 9 p.m) and 8.56 pg/ml (at 9 a.m). Plasma melatonin concentrations started to rise at 9.22 p.m with highest peak (74.91 pg/ml) at 2.55 a.m.

**Figure 4:**
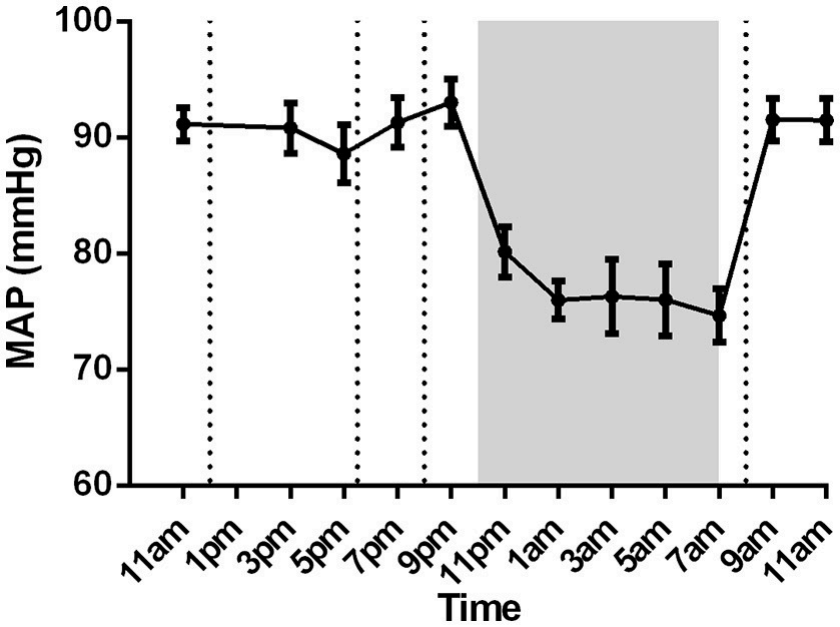
Mean arterial pressure of nine healthy male subjects (mean ± SEM). Lunch, dinner, evening snack and breakfast are marked with dotted lines in respective order. Night-time (lights off at 10 p.m; lights on at 7 a.m) is marked with shaded region.

Mean arterial pressure showed circadian rhythm with higher pressure during daytime compared to night time (Figure 4).

## Discussion

OXA affects wake/sleep cycle and feeding. In the present study we established a sensitive OXA RIA and demonstrated that plasma levels were low but detectable, and did not relate to sleep/wake rhythm or feeding while melatonin and cortisol levels followed their known circadian rhythms.

OXA and/or its receptor(s) have been detected in peripheral tissues such as adrenal gland, pancreas, and testis (1, 28–31). Yet, the functions of the peptide/receptors have not been elucidated. Besides, low circulating levels of OXA without circadian rhythm do not strongly support a physiological role in periphery.

There are different RIAs and ELISAs to measure OXA in human and animal plasma. The measured concentrations, however, vary greatly between the studies depending on study design and methodologies, ranging from lower than 1 pg/ml to about 2,500 pg/ml (13, 19, 20, 25, 32–37). In addition, HPLC separations of plasma extracts have showed two or more immunoreactive peaks (21, 35). The great variability could thus be explained by the recognition of multiple epitopes of the antiserum and/or use of unpurified/unprocessed samples. In the present study, we used solid phase extraction of plasma samples in combination with a highly sensitive and specific RIA for detecting human plasma OXA concentrations. We also showed that the immunoreactivity of the solid phase extract eluted as synthetic OXA and no other immunoreactive peaks were observed. This has not been observed in the previous studies. The daily plasma OXA concentration varied between undetectable levels from 0.5 to −16 pg/ml. Importantly, the peptide concentrations were below detection limit of the RIA in two of our subjects.

In CSF, OXA has been shown to follow circadian rhythm with high concentrations late at night (3, 9). However, the differences between day- and night values observed were minor, from 4 to 9% depending on the study. Several studies have demonstrated that orexin is responsible for the stabilization of wakefulness (2, 5, 6). Strawn et al. also suggested that CSF OXA correlates with plasma OXA (37). However, the plasma OXA concentrations were as high as 2.5 ng/mL. These results were obtained via C18 extracted samples with a commercial ELISA kit and are at the highest end of reported values. It should be noted that OXA concentrations in naïve CSF samples are about 200–400 pg/ml in healthy subjects but close to 100 pg/ml in subjects with narcolepsy (40). It is therefore not probable that plasma OXA concentrations could exceed tenfold those in CSF.

Whether the failure of the orexin system of the narcoleptic patients reflects also changes in plasma OXA levels is currently unknown. Higuchi et al. reported significantly lowered plasma OXA concentrations in narcoleptic patients, while Dalal et al. using an RIA kit from a different vendor did not find any differences between control and patient samples (4, 33). In both studies, values were near the detection limits of their assays making the assessment of the results challenging to interpret (4, 33, 41). Recently, Tang et al. (13) presented a positive correlation between plasma OXA concentrations and the Insomnia Severity Index (13); their measured OXA concentrations were 55 pg/ml, higher than any plasma concentrations found in our healthy male subjects. In our study, we measured plasma OXA concentrations from healthy male subjects during a 24 h cycle. Our results showed that plasma OXA did not associate to wake/sleep states. As control, all subjects showed both nocturnal melatonin secretions as well as cortisol fluctuations with early morning peak. Mean arterial pressure was also lowest at night. The presence of these normal circadian rhythms show that the blood sampling through catheters did not interfere sleep/wake cycle. However, the number of participants was low and the difference in the OXA concentrations in a daily cycle could have been lost.

Orexins are known for their actions on both short- and long-term feeding. Intra-cerebroventricular injection of OXA induces feeding in rodents (14–16). Similarly, chronic administration of OXA affects feeding in rats (16, 17). Blocking OXA-mediated functions with intra-peritoneally applied OX1R antagonist or intra-cisternally applied OXA antibody resulted in reduced food intake in fasted rats (42, 43). Orexins may also act as a link between metabolic state and arousal (44). Isolated mouse orexin neurons are inhibited by administration of glucose and leptin. In addition, fasting promotes wakefulness in wild type mice, but not in orexin neuron-ablated mice. These findings indicate that orexin neurons might sense metabolic signals arising from the periphery and maintain wakefulness during negative energy balance. In the present study, no significant differences were observed in solid phase extracted OXA plasma concentrations related to meals. Depending on the subject, the peptide levels showed an increase, decrease or no change after meals. In one subject one major peak lasting over the next meal was observed after dinner. Thus, acute feeding does not seem to affect OXA plasma concentrations in our healthy subjects.

We demonstrated using a sensitive RIA and solid phase extraction of plasma samples that OXA is detectable in low concentrations in most human samples, and not modulated by feeding or sleep/wake cycle in healthy male subjects. OXA is produced throughout the CNS and can cross the blood brain barrier (45). OXA concentrations in CSF are much higher than that detected in the present study in the plasma and it is possible that low amounts of OXA could diffuse from brain tissues or CSF to the plasma. However, due to low levels and high individual variations in healthy subjects it is unclear if plasma orexin has any physiological significance as related to autonomic and neuroendocrine rhythms. The role of OXA in pathological states involving the blood brain barrier (such as old age, Alzheimer's disease and multiple sclerosis) needs to be investigated in further studies.

## Author Contributions

KM, JL and K-HH designed the study, acquired the data, took (K-HH) and processed the blood samples, and analyzed the samples. TK and AJA contributed to the development of OXA RIA. OV did the analysis of melatonin and contributed to revision of the manuscript. KM drafted the first version of the manuscript. All authors contributed and approved the final version of the manuscript.

### Conflict of Interest Statement

The authors declare that the research was conducted in the absence of any commercial or financial relationships that could be construed as a potential conflict of interest.
